# The Novel Compound Heterozygous Mutations of *ECEL1* Identified in a Family with Distal Arthrogryposis Type 5D

**DOI:** 10.1155/2020/2149342

**Published:** 2020-05-23

**Authors:** Jie-Yuan Jin, Dan-Yu Liu, Zi-Jun Jiao, Yi Dong, Jie Li, Rong Xiang

**Affiliations:** ^1^School of Life Sciences, Central South University, Changsha, China; ^2^Department of Respiratory and Critical Care Medicine, Peking University First Hospital, Beijing, China; ^3^Department of Emergency, Peking Union Medical College Hospital, Chinese Academy of Medical Sciences and Peking Union Medical College, Beijing, China; ^4^Human Key Laboratory of Animal Models for Human Diseases, School of Life Sciences, Central South University, Changsha, China

## Abstract

**Introduction:**

Distal arthrogryposis type 5D (DA5D) is an autosomal recessive disease. The clinical symptoms include contractures of the joints of limbs, especially camptodactyly of the hands and/or feet, unilateral ptosis, a round-shaped face, arched eyebrows, and micrognathia, without ophthalmoplegia. *ECEL1* is a DA5D causative gene that encodes a membrane-bound metalloprotease. ECEL1 plays important roles in the final axonal arborization of motor nerves in limb skeletal muscles and neuromuscular junction formation during prenatal development.

**Methods:**

A DA5D family with webbing of the elbows and fingers was recruited. We performed whole-exome sequencing (WES) and filtered mutations by disease-causing genes of arthrogryposis multiplex congenita (AMC). Mutational analysis and cosegregation confirmation were then performed.

**Results:**

We identified novel compound heterozygous mutations of *ECEL1* (NM_004826: c.69C>A, p.C23∗ and c.1810G>A, p.G604R) in the proband.

**Conclusions:**

We detected causative mutations in a DA5D family, expanding the spectrum of known *ECEL1* mutations and contributing to the clinical diagnosis of DA5D.

## 1. Introduction

Arthrogryposis multiplex congenita (AMC) is a heterogeneous group of disorders characterized by multiple congenital nonprogressive joint contractures, usually involving the limbs [1]. When contractures mainly involve the distal joints and affect the hands, feet, wrists, and ankles, the disease is named distal arthrogryposis (DA) [2]. DA is phenotypically and genetically heterogeneous, can be divided into at least 10 different forms (DA1–DA10), and involves more than 9 causative genes, including *TPM2*, *TNNI2*, *TNNT3*, *MYH3*, *MYBPC1*, *MYH8*, *FBN2*, *PIEZO2*, and *ECEL1* [3–6].

Of all the DA syndromes, DA5D is perhaps the most notable, because of its unique phenotype, comprising camptodactyly of the hands and/or feet, clubfoot and/or a calcaneovalgus deformity, extension contractures of the knee, unilateral ptosis, a round-shaped face, arched eyebrows, a bulbous upturned nose, and micrognathia [6]. Unlike other DAs inherited with autosomal dominant heredity (except rare families segregating recessive disease-causing *PIEZO2* variants), DA5D is an autosomal recessive disease [7]. Compound heterozygous or homozygous mutations of *ECEL1* can cause DA5D [6].

*ECEL1* is located on 2q36-q37, encoding endothelin-converting enzyme-like 1, a membrane-bound metalloprotease. In contrast to most causal genes of DA encoding proteins related to the muscle contraction apparatus, *ECEL1* is predominantly expressed in neuronal tissue from embryonic stages [8]. A mouse model showed that ECEL1 functional disruption leads to impaired axonal arborization of the motor nerves in limb muscles and results in subsequent neuromuscular junction (NMJ) formation failure, suggesting that DA with *ECEL1* mutations could result from neurogenic pathogenesis through developmental defects of the presynaptic motor nerves in the limbs, although the detailed molecular mechanism remains unknown [9, 10].

In the present study, we reported a family with DA5D from Hunan Province, China. We identified novel compound heterozygous mutations of *ECEL1* (NM_004826: c.69C>A, p.C23∗ and c.1810G>A, p.G604R) in the proband, which were inherited from his parents. To the best of our knowledge, this group of mutations has not been previously reported.

## 2. Materials and Methods

### 2.1. Patients and Subjects

The Review Board of Xiangya Hospital of Central South University approved this research. Written informed consent was obtained from the patient and his guardians, in which all subjects consented to this study and the publication of the images. Blood was collected from the proband and related family members. Segregation analysis was performed in all family members based on the whole-exome sequencing (WES) results.

### 2.2. Whole-Exome Sequencing

Genomic DNA was extracted with the DNeasy Blood and Tissue Kit (Qiagen, Valencia, California, USA). The Novogene Bioinformatics Institute (Beijing, China) provided exome capture, high-throughput sequencing, and common variant filtering. All the exomes were captured using the Agilent SureSelect Human All Exon V5 Kit and sequenced using the Illumina HiSeq2000 platform. After filtering the common variants (frequency ≥ 0.05) using the 1000 Genomes Project database (https://www.genome.gov/27528684/1000-genomes-project/), the Chinese Millionome Database (https://db.cngb.org/cmdb/), the Genome Aggregation Database (http://gnomad.broadinstitule.org), and the Exome Aggregation Consortium database (http://exac.broadinstitute.org/), unique single-nucleotide polymorphisms (SNPs) were detected in subjects. Potential causative variants were screened by the list of genes related to AMC [1, 11] (Table S1) and then predicted by bioinformatics programs including MutationTaster (http://www.mutationtaster.org/), Polyphen-2 (http://genetics.bwh.harvard.edu/pph2/), and SIFT (http://provean.jcvi.org/index.php). The analyses of gene function, inheritance pattern, and clinical phenotype were conducted using Online Mendelian Inheritance in Man (OMIM) (https://www.omim.org).

### 2.3. Sanger Sequencing

Primer pairs were designed by DNASTAR, and the sequences of primers will be provided upon request. The target fragments were amplified with polymerase chain reaction (PCR) and analyzed using the ABI 3100 Genetic Analyzer (ABI, Foster City, CA).

## 3. Results

### 3.1. Clinical Features

**Figure 1:**
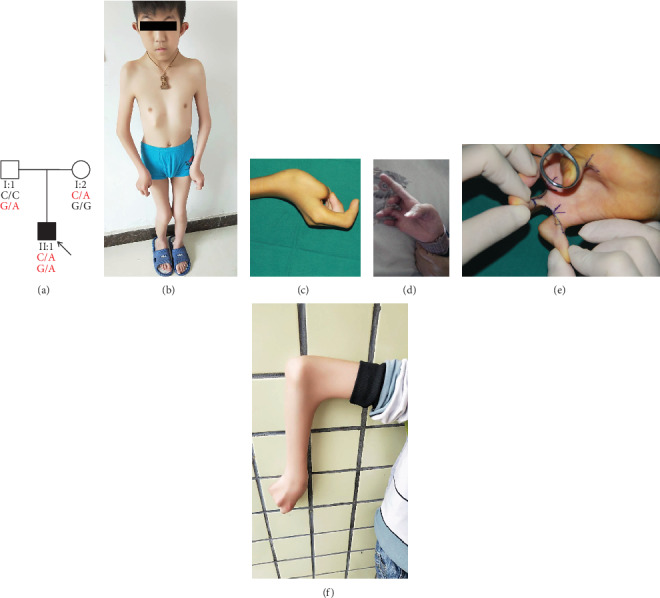
(a) Pedigree of the DA5D family with segregation analysis. The black symbols represent an affected member, and the arrow indicates the proband. Genotypes are identified by letters and slash, with red representing mutations. (b–f) The parts of phenotypes of the proband. (b) Facial features and contractures of the wrists, elbows, and knees in the proband. The proband had the bilateral distal arthrogryposis (c, d) and the webbing of bilateral fingers (e) and elbows (f).

We identified a DA5D family from Hunan Province, China (Figure 1(a)). The proband (II:1) was a 10-year-old boy. He was born at term, with a birth weight of 3.4 kg. His height was 135 cm (14%), and his weight was 21 kg (<0.5%). The patient had bilateral contractures of the fingers, wrist, elbow, and knees, without abnormal feet (Figures 1(b)–1(d)). Notably, he had webbing of the bilateral fingers and elbows (Figures 1(e) and 1(f)). He presented facial features, including left mild facial weakness, left ptosis, arched eyebrows, strabismus, protruding ears, and cleft palate (surgically repaired at the age of 3 years) (Figure 1(b)). He suffered from cryptorchidism and ventral hernia. His psychomotor development was within normal limits. His parents (I:1 and I:2) were not affected.

### 3.2. Genetic Analysis

WES yielded 9.12 GB of data with 99.6% coverage of the target region and 99.0% of the target covered over 10×. After a series of database analyses and filtering, 1118 unique SNPs were detected in the proband. Variants were filtered by AMG genes (Table S1), and a set of six variants in five genes in the patient was identified (Table 1). By analyzing the bioinformatics prediction, inheritance pattern, OMIM clinical phenotypes, and American College of Medical Genetics classification [12] of these six variants, we highly suspected the mutations (NM_004826: c.69C>A, p.C23∗ and c.1810G>A, p.G604R) of *ECEL1* to be the genetic lesions of the patient.

**Figure 2:**
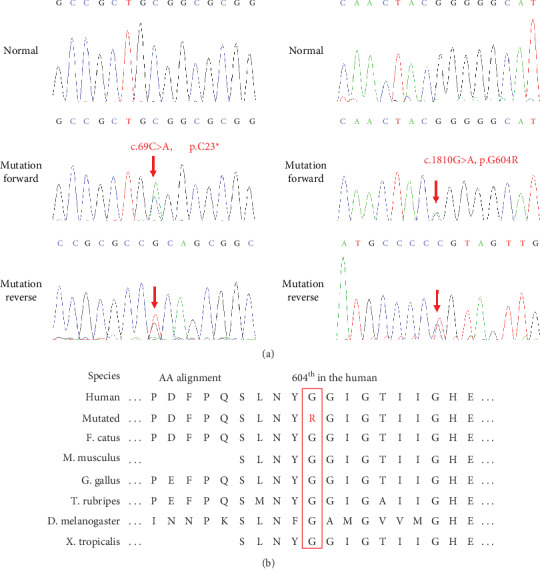
(a) Sequencing results of the *ECEL1* mutations. Sequence chromatograms indicate compound heterozygosity (NM_004826: c.69C>A, p.C23∗ and c.1810G>A, p.G604R) in the family with DA5D. (b) The mutation sites (G604R) are highly evolutionarily conserved across species. The red graph represents mutated amino acids, and the red box emphasizes these sites across species for comparison.

Sanger sequencing showed that the novel nonsense mutation (c.69C>A, p.C23∗) of *ECEL1* in the patient was inherited from his mother, and another mutation (c.1810G>A, p.G604R) was from his father; each cosegregated with the affected family member (Figure 2(a)). Additionally, the amino acid sequence alignment analysis suggested that the mutation (c.1810G>A, p.G604R) was located in a highly evolutionarily conserved site (Figure 2(b)).

## 4. Discussion

DA5 is distinguished from other forms of DA by the presence of ocular abnormalities, typically ptosis, ophthalmoplegia, and/or strabismus, in addition to contractures of the distal joints [13]. Furthermore, some affected individuals develop restrictive lung disease with resultant hypoxemia, hypercarbia, pulmonary hypertension, and early death [6]. McMillin et al. designated DA5D as a subset of DA5 and identified the causative gene, *ECEL1* [6]. Unlike typical DA5, the inheritance of DA5D is an autosomal recessive form, and the patients do not have ophthalmoplegia. Our patient suffered from distal joint contractures, unilateral ptosis, and strabismus, without ophthalmoplegia, and molecular genetics testing confirmed his compound heterozygous *ECEL1* mutations. All evidence supported that the patient had DA5D. Our patient presented webbing of the elbows and fingers, which are very infrequent (we only found one case in each type, both reported by McMillin et al.) [6]. It must be noted that Dohrn et al. reported two aborted fetuses, whose gestational ages at termination of pregnancy were 14 + 2 weeks and 13 + 4 weeks, with the homozygous *ECEL1* mutation having fixated flexion and webbing in the elbows [14]. Additionally, the proband had malnutrition not caused by ingestion of nutrition. Our description broadens the clinical spectrum of DA5D.

*ECEL1* encodes a type II integral transmembrane zinc metalloprotease, whose exact substrate remains unknown. Despite its structural similarity to endothelin-converting enzyme (ECE), ECEL1 does not cleave ECE substrates [15]. *In vitro* studies of transfected cells suggested that it localizes to the endoplasmic reticulum and to a lesser extent to the cell surface [16]. The rodent homologue of *ECEL1* is termed damage-induced neuronal endopeptidase (*DINE*). In mice, DINE is significantly upregulated in both the peripheral and central nervous systems in response to various neuronal injuries and plays essential roles in final axonal arborization of motor nerves in the limb skeletal muscles and the formation of proper NMJs during prenatal development [9]. Indeed, DINE/ECEL1-deficient mice die immediately after birth due to respiratory failure [17]. Pterygia or webs are found across joints, reflecting an early and sustained lack of movement during in utero development, which is often related to failure of formation and maturation of the embryonic neuromuscular end-plate [1]. The webbing of fingers and elbows in our patient suggested that his clinical symptoms (at least part of his symptoms) were caused by neuromuscular end-plate formation failure, which is consistent with the phenotypes of ECEL1-deficient mice. In fact, DA5D patients with pterygia are not unusual [14].

**Figure 3:**
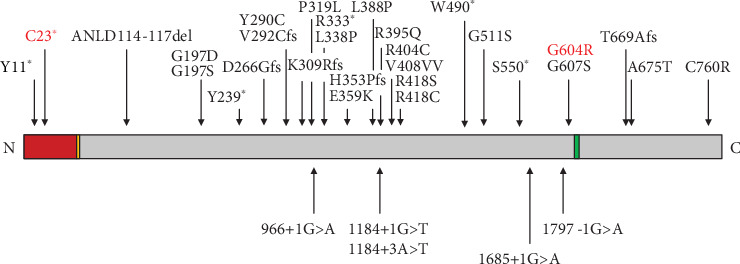
*ECEL1* mutations identified in DA5D patients. The rectangular box represents the ECEL1 protein with the N-terminus on the left and C-terminus on the right. Known functional domains include a cytoplasmic domain (indicated by the red box), a transmembrane domain (yellow box), and an extracellular domain (gray box) with a zinc binding motif (green box). Vertical arrows show the approximate location of mutations of *ECEL1*. The red words represent previous mutations.

There are at least 34 mutations in *ECEL1* [2, 6, 7, 18, 19] (Figure 3). The structure of ECEL1 can be roughly divided into a cytoplasmic domain, a transmembrane domain, and an extracellular domain with a zinc binding motif (the 612^th^-616^th^ AA) essential for enzymatic activity [6, 7]. The nonsense mutation in our patient (c.69C>A, p.C23∗) occurred in the cytoplasmic domain, producing a premature terminated protein. The missense mutation (c.1810G>A, p.G604R) occurred near the activation site, potentially affecting biological function. Shaaban et al. reported a mutation (c.1810G>A, p.G607S) adjacent to G604R in a DA5D family, and Kiryu et al. confirmed the loss of function in homozygous G607S mutant mice [18, 19]. G604R may have a similar pathogenicity. The variant (c.1810G>A) was reported in the ClinVar, ExAC, and gnomAD databases, without the homozygous state. Given that a single heterozygous *ECEL1* mutation did not lead to DA5D, it is natural that a causative variant was detected in a few people. The disease in our patient results from a combination of two mutations.

## 5. Conclusions

In summary, we used WES to explore the genetic factors in a Chinese family with DA5D. Novel compound heterozygous mutations of *ECEL1* (NM_004826: c.69C>A, p.C23∗ and c.1810G>A, p.G604R) were detected and coseparated in the family members. Our description expands the spectrum of known *ECEL1* mutations and contributes to the clinical diagnosis of DA5D.

## Figures and Tables

**Table 1:** Variants identified by WES in combination with AMC-related gene-filtering in the present patient.

Gene	Variant	Mutation taster	PolyPhen-2	SIFT	1000G	ExAC	gnomAD	OMIM clinical phenotype	American College of Medical Genetics classification^∗^
*NEB*	NM_001164508: c.19295A>G, p.Q6432R	D (0.872)	D (0.985)	T (0.103)	0.0016	0.0005	0.0004	AR; nemaline myopathy 2	PP3, BP5
*ECEL1*	NM_004826: c.1810G>A, p.G604R	D (1.000)	D (1.000)	D (0.000)	—	0.0000	0.0001	AR; arthrogryposis, distal, type 5D	PM2, PP3, PP4
*ECEL1*	NM_004826: c.69C>A, p.C23∗	D (1.000)	—	—	—	—	—		PVS1, PM2, PP3, PP4
*CD96*	NM_198196: c.901A>G, p.I301V	P (1.000)	B (0.089)	D (0.026)	—	0.0001	0.0000	AD; C syndrome	PM2, BS4, BP4, BP5
*SCARF2*	NM_182895: c.1796C>T, p.A599V	P (0.990)	D (0.351)	D (0.024)	—	—	—	AR; Van den Ende-Gupta syndrome	PM2, PP3, BP5
*FGD1*	NM_004463: c.1340+9C>T	D (1.000)	—	—	0.0032	0.0008	0.0005	XLR; Aarskog-Scott syndrome/XLR; mental retardation, X-linked syndromic 16	BP4, BP5

D: disease causing; T: tolerated; P: polymorphism; B: benign; AR: autosomal recessive; AD: autosomal dominant; XLR: X-linked recessive. ^∗^Pathogenic: PVS1> PS1> …> PS4> PM1-6> PP1-5; benign: BA1> BS1-4> BP1-7. PVS: pathogenic very strong; PS: pathogenic strong; PM: pathogenic moderate; PP: pathogenic supporting; BA: benign stand alone; BS: benign strong; BP: benign supporting.

## Data Availability

No additional data are available.

## Supplementary Materials

Supplementary Materials Table S1 lists 191 genes which were identified in patients with arthrogryposis multiplex congenita (AMC). This is a panel of AMC genes. We screened variants in this study by it.
