# Supplementary material for: The Novel Compound Heterozygous Mutations of ECEL1 Identified in a Family with Distal Arthrogryposis Type 5D
Source: Biomed Res Int. 2020 May 23;2020:2149342. doi: 10.1155/2020/2149342 (PMC7273484; doi:10.1155/2020/2149342)
Supplement: Supplementary Materials — Table S1 lists 191 genes which were identified in patients with arthrogryposis multiplex congenita (AMC). This is a panel of AMC genes. We screened variants in this study by it. [file 2149342.f1.pdf]

Supplementary Table 1. Causative/potential causative genes of AMC.

ACTA1	*ADAMS10*	*ADAMTSL2*	*ADCY6*	*ADGRG6*	*ADSL*	*AKT1*
ANTXR2	*AP1S2*	*ARX*	*ASXL1*	*ATRX*	*B3GAT3*	*BIN1*
CANT1	*CASK*	*CATSAL*	*CATSHL*	*CD96*	*CHAT*	*CHRNA1*
CHRNB1	*CHRND*	*CHRNE*	*CHRNG*	*CHST14*	*CMG2*	*CNTN1*
CNTNAP1	*COFS*	*COL1A1*	*COL1A2*	*COL1A5*	*COL2A1*	*COL6A1*
COL6A2	*COL6A3*	*COL7A1*	*COLEC10*	*COLEC11*	*CRLF1*	*DCX*
DHCR7	*DNM2*	*DOK7*	*DRG2*	*DTDST*	*DYM*	*EBP*
ECEL1	*EIF2S3*	*EMG1*	*ERBB3*	*ERCC1*	*ERCC2*	*ERCC5*
ERCC6	*ERG2*	*ESCO2*	*FBN1*	*FBN2*	*FCMD*	*FGD1*
FGF9	*FGFR1*	*FGFR2*	*FGFR3*	*FHL1*	*FKBP10*	*FKRP*
FKTN	*FLNA*	*FLNB*	*FLVCR2*	*GBA*	*GDF5*	*GJA1*
GLE1	*GLI3*	*GLRA1*	*GLRB*	*GPC3*	*GRUL3*	*HOXA13*
HOXD13	*HSPG2*	*IMPAD1*	*INSR*	*IRF6*	*KAT6B*	*KCNA1*
KIAA0196	*KIF7*	*LARGE*	*LICAM*	*LIS1*	*LMBR1*	*LMX1B*
MASP1	*MED12*	*MHS3*	*MMP2*	*MNX1*	*MPZ*	*MTM1*
MUSK	*MYBPC1*	*MYH3*	*MYH8*	*NEB*	*NF1*	*NOG*
NSD1	*OFD1*	*PAFAH1B1*	*PAX3*	*PEHO*	*PEX1*	*PEX12*
PEX14	*PEX2*	*PEX26*	*PEX3*	*PEX5*	*PEX6*	*PEX7*
PFKM	*PIEZO2*	*PIP5KIC*	*PLOD1*	*PLOD2*	*PM11*	*PMM2*
PMP22	*PMPK*	*POMGNT1*	*POMT1*	*POMT2*	*POR*	*PRG4*
PRX	*PTDSS1*	*PTHR*	*PTLAH*	*RAB18*	*RAB3GAP1*	*RAB3GAP2*
RAPSN	*RBM10*	*RELN*	*RET*	*RIPK4*	*RNASEH2A*	*RNASEH2B*
RNASEH2C	*RYR1*	*SAMHD1*	*SCARF2*	*SELENON*	*SEPN2*	*SETBP1*
SHOX	*SLC05A1*	*SLC26A2*	*SLC9A6*	*SLCO5A1*	*SMARCAD1*	*SMN*
SOX9	*SULF1*	*SYNE1*	*TARP*	*TBX22*	*TBX5*	*TNNI2*
TNNT3	*TPM2*	*TPM3*	*TREX1*	*TRPV4*	*TSC1*	*TSC2*
UBA1	*UBE1*	*UPK3A*	*VIPAR*	*VPS33B*	*WNT7A*	*ZBTB42*
ZC4H2	*ZIC3*					
